# Non-uremic Calciphylaxis With Possible Initial Misdiagnosis as Erythema Multiforme/Toxic Epidermal Necrolysis Successfully Treated as Inpatient

**DOI:** 10.7759/cureus.43618

**Published:** 2023-08-17

**Authors:** Raed Al yacoub, Faread Jamalifard, Michael Ladna, Addie Walker

**Affiliations:** 1 Internal Medicine, University of Florida College of Medicine, Gainesville, USA; 2 Medicine, University of Florida College of Medicine, Gainesville, USA; 3 Dermatopathology, University of Florida College of Medicine, Gainesville, USA

**Keywords:** toxic epidermal necrolysis, erythema multiforme, case report, non-uremic calciphylaxis management, non-uremic calciphylaxis diagnosis

## Abstract

Non-uremic calciphylaxis (NUC) is a rare, high-mortality disease, and it can be easily misdiagnosed as other ulcerative dermatologic conditions. A female in her late 30s with a medical history of alcoholic liver cirrhosis and obesity who previously underwent gastric bypass surgery presented with an 11-month history of worsening bilateral lower extremity wounds following the initiation of spironolactone. A wound biopsy at the time of initial presentation favored erythema multiforme/toxic epidermal necrolysis (EM/TEN). She initially responded to systemic steroids, but her wounds later worsened, prompting her to seek representation a few months later. The initial suspicion was for a superimposed bacterial infection; however, her wounds did not improve following antibiotics. A repeat skin biopsy revealed calciphylaxis, for which she had multiple risk factors, including severe vitamin D deficiency causing secondary hyperparathyroidism. A multidisciplinary approach was successful in achieving a satisfactory response with pain control, wound care, skin grafting, and mitigation of risk factors in addition to the use of sodium thiosulfate. Upon our review, the initial biopsy did not demonstrate features of EM/TEN but did demonstrate features suspicious for calciphylaxis. The exposure to systemic corticosteroids due to the presumed diagnosis of EM/TEN may have worsened her condition since this is a known risk factor for calciphylaxis. Our case highlights the importance of clinicopathologic correlation as well as the place for calciphylaxis in the clinical and histopathologic differential diagnosis of ulcerated, necrotic lesions on the lower extremities in the absence of renal disease.

## Introduction

Non-uremic calciphylaxis (NUC) is a high-mortality skin condition occurring in patients without medical renal disease or dialysis therapy [1]. The data are limited to small retrospective studies and case series, as it is not a common condition. A systematic review of 36 cases of non-uremic calciphylaxis in 2008 by Nigwekar et al. reported the most common risk factors of calciphylaxis, including primary hyperparathyroidism, malignancy, alcoholic liver disease, and connective tissue disease [2]. Warfarin and corticosteroid use were seen in 25% and 61% of patients, respectively [2]. Mortality was 52%, with sepsis being the leading cause of death [2]. Case reports have also described calciphylaxis as a complication of Hodgkin lymphoma [3], teriparatide therapy [4], gastric bypass [5], and hypoparathyroidism [6].

Due to the rarity of non-uremic calciphylaxis, it can be misdiagnosed incorrectly as ulcerative dermatologic conditions such as infection, vasculopathy, vasculitis, necrotizing fasciitis, or pyoderma gangrenosum. It is critical that the correct diagnosis be made in a timely fashion due to the high one-year mortality associated with calciphylaxis. NUC can be exacerbated by systemic corticosteroids, the therapy of choice for many of the similar-appearing ulcerative skin pathologies. Therapeutic options are not well studied and are limited to retrospective case series and case reports. This case report illustrates a classic example of the rare entity of NUC in a patient with several known risk factors who likely was initially misdiagnosed with erythema multiforme/toxic epidermal necrolysis (EM/TEN) following an initial histopathologic review and was subsequently successfully treated via surgical debridement and sodium thiosulfate.

## Case presentation

A female in her late 30s presented to the hospital with worsening chronic bilateral lower extremity painful wounds. The wounds initially appeared 11 months prior to admission, following the initiation of spironolactone. A punch biopsy measuring 1.7 cm × 1.0 cm × 0.9 cm was performed, with a clinical history of "lesions and blisters to the lower legs" supplied on the pathology requisition form. The histopathologic diagnosis favored EM/TEN. Spironolactone was discontinued, and she was treated with prednisone with improvement. She was discharged to a nursing facility and received rigorous wound care; however, her wounds never completely resolved. A few months later, she was admitted to the hospital for worsening of her wounds and concern for a superimposed infection. She completed a course of antibiotics without significant improvement and was transferred to our hospital for further management, including a dermatology evaluation. A physical exam revealed diffuse bilateral lower extremity edema with chronic non-healing wounds and deep necrotic black eschars surrounded by firm, violaceous, non-blancheable patches most prominent over her anterior thighs. Her past medical history is significant for acquired hypothyroidism due to multinodular goiter status post-thyroidectomy, alcoholic liver cirrhosis, and obesity status post-bariatric surgery.

Laboratory investigation in Table 1 revealed albumin 2.3 g/dL (normal 3.5-5.2 g/dL), total calcium 7.4 mg/dL (normal 8.4-10.2 mg/dL), within normal limit ionized calcium, phosphorus 2.2 mg/dL (normal 2.7-4.5 mg/dL), alkaline phosphatase 367 IU/L (33-133 IU/L), parathyroid hormone 89 (normal 12-88 pg/mL), and vitamin D 25-hydroxy <7 ng/mL (normal 20-120 ng/mL). Anti-B2 glycoprotein IgM 9.4 smuu/mL (normal <20 sguu/mL) with all other antiphospholipid syndrome antibodies negative. Coagulation testing was normal including Protein C and S activity. The full blood count, creatinine, and blood urea nitrogen were within normal limits. Anti-nuclear antibody and anti-neutrophil cytoplasmic antibody testing were negative. Punch biopsy of 6 mm diameter and 6 mm depth of the left thigh wound revealed fat necrosis with subcutaneous vessel wall calcification consistent with calciphylaxis. Epidermal changes of EM/TEN were not present. Tissue cultures yielded no growth of fungal, bacterial, or acid-fast organisms.

**Table 1:** Lab values g: gram, dL: deciliter, mg: milligram, ml: milliliter, mmol: millimole, IU: international unit, pg: picogram, ng: nanograms, smu: source measure unit.

Lab results		Normal range
Albumin	2.3 g/dL	3.5–5.2 g/dL
Total calcium	7.4 mg/dL	8.4-10.2 mg/dL
Ionized calcium	1.3 mmol/L	1.15 mmol/L
Phosphorus	2.2 mg/dL	2.7–4.5 mg/dL
Alkaline phosphatase	367 IU/L	33–133 IU/L
Parathyroid hormone	89 pg/mL	12–88 pg/mL
Vitamin D 25-hydroxy	<7 ng/mL	20–120 ng/mL
Anti-B2 glycoprotein IgM	<9.4 smu/mL	<20 smu/mL
ANA	<1:80	<1:80
Anti-neutrophil cytoplasmic antibody	<1:20	<1:20
Protein C activity	80%	>65%
Protein S activity	95.4%	63.5–149%

The clinical differential diagnosis included uremic calciphylaxis, which was ruled out in the absence of end-stage renal disease (ESRD) or renal insufficiency. Erythema multiforme/toxic epidermal necrolysis was considered given the previous diagnosis; however, no evidence of this entity was found on a punch biopsy. Additionally, vasculitis, alternative vasculopathy, and rheumatologic processes, including antiphospholipid syndrome, were considered; however, biopsy and serologic investigation were unrevealing other than an isolated elevated serum anti-B2 glycoprotein IgG without a proven vasculopathic event. Given the exam findings and the punch biopsy result, the patient was diagnosed with NUC.

With consensus recommendation by nephrology and dermatology, this patient was started on IV sodium thiosulfate 25 g daily, later decreased to three times weekly due to the development of metabolic acidosis (bicarbonate in the metabolic panel was 18; normal range 22-30 MMOL/L). Following the initiation of sodium thiosulfate, she was taken to the operating room multiple times by the burn surgical team for wound debridement, application of allografts, and wound vacuum-assisted closure (VAC) placement or exchange. She received aggressive wound care. Endocrinology evaluated her severe vitamin D deficiency with resultant secondary hyperparathyroidism and hypophosphatemia, recommending the initiation of oral ergocalciferol at 50,000 IU weekly.

**Figure 1:**
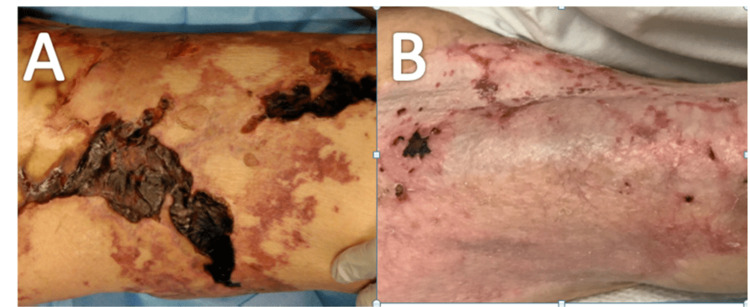
Image of right hip before and after treatment Right hip lesion on presentation (A) and right hip lesion s/p sodium thiosulfate and grafting (B).

The patient in total received IV sodium thiosulfate over 39 days. At the time of discontinuation, wound VACs had been removed, with wounds well healing, demonstrating good vascularization, and no evidence of necrosis (Figure 1). She continued to receive aggressive wound care. She was discharged to a skilled nursing facility and provided outpatient follow-up with both dermatology and burn surgery.

Later, the initial biopsy slides and pathology report were obtained from the original facility and reviewed. The histopathology shows epidermal necrosis with subepidermal blister formation but without the pattern of interface change and keratinocyte necrosis characteristic of EM/TEN. Ample subcutaneous fat demonstrates large deep vessels with dystrophic calcifications as well as smaller vessels with associated small deposits of material, which could raise suspicion for calciphylaxis-type calcifications.

## Discussion

Calciphylaxis is a serious, rare disorder resulting in skin ischemia and necrosis, causing severely painful skin lesions that demonstrate poor healing and are frequently complicated by blistering, ulceration, and infection [7,8]. Histologically, it is characterized by calcification of arterioles and capillaries in the deep dermis and subcutaneous adipose tissue [7]. Calciphylaxis has a high one-year mortality rate of 45% to 80%, with ulcerated lesions having higher mortality than non-ulcerated lesions [1]. Sepsis is the leading cause of death [1]. A definitive diagnosis requires a skin biopsy, with incisional or punch biopsy sampling of ample subcutaneous adipose tissue being the preferred route. Biopsy should be from the lesion margin since the center of the ulcer or necrotic area tends to be of low diagnostic yield [1].

Calciphylaxis primarily affects patients who have chronic kidney disease or end-stage renal disease treated by dialysis, referred to as uremic calciphylaxis, and rarely may affect patients with normal kidney function, referred to as non-uremic calciphylaxis.

**Figure 2:**
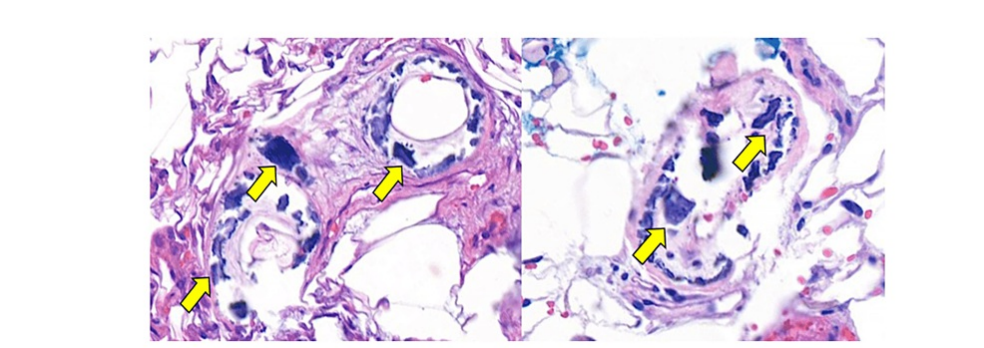
Skin biopsy of the thigh Punch biopsy of left thigh with vessel wall calcifications (arrows) in subcutaneous adipose tissue (hematoxylin and eosin, 100×).

Calciphylaxis is observed in our patient due to a constellation of multiple risk factors. These factors encompass a history of high-dose corticosteroid therapy, post-Roux-en-Y gastric bypass obesity, alcoholic liver disease, hypoalbuminemia, severe Vitamin D deficiency resulting in secondary hyperparathyroidism, as well as female gender. Her skin biopsy showed fat necrosis with subcutaneous vessel wall calcification, as seen in Figure 2. A review of the initial biopsy indicated that a specimen of adequate size and depth was taken. However, a relatively uninformative clinical history of "lesions and blisters" was given to the pathologist. The resulting histopathologic diagnosis of EM/TEN was likely incompatible with the clinical presentation, and an opportunity for clinicopathologic correlation and discussion between the clinical team and pathology may have been overlooked. It is the writer’s opinion that the patient’s wounds were likely secondary to NUC all along, and thus her potential unnecessary exposure to systemic corticosteroids added another risk factor for NUC and may have contributed to the secondary worsening of her wounds.

The therapeutic approach for calciphylaxis must be multidisciplinary to achieve any form of success, including pain control, wound care, and mitigation of risk factors, in addition to the use of medications of uncertain efficacy, including sodium thiosulfate, bisphosphonates, and calcimimetics [1,9].

The use of sodium thiosulfate is better studied in hemodialysis patients. In a systematic review of 172 HD patients with calciphylaxis, 47.2% reported improvement, and 26.4% reported complete resolution of calciphylaxis with sodium thiosulfate [9]. The mechanism of action of sodium thiosulfate is contested; originally thought to be a calcium chelating effect, recent data points towards direct vascular calcification inhibition, an antioxidant effect, and a vasodilatory effect [10]. The duration of treatment is unclear; however, expert opinion suggests improvement in pain within one to two weeks following initiation is a predictor of long-term response [1]. Expert opinion advises maintaining normal serum calcium and phosphorus levels and a parathyroid hormone (PTH) level between 150 and 300 [1]. Cinacalcet is the preferred treatment for secondary hyperparathyroidism over vitamin D analogues in calciphylaxis [11]. Other treatments based on case reports and case series include hyperbaric oxygen therapy and sterile maggot therapy with larvae of the greenbottle fly, Lucilia sericata, which are being used in certain centers [12,13]. Nigwekar et al. also reported a reduced risk of calciphylaxis associated with statin use [14].

Data on the treatment of nonuremic calciphylaxis is very limited. Sodium thiosulfate is the first-line therapy based on available data [15]. Our patient received aggressive wound care, skin grafting, pain management, and treatment of underlying severe vitamin D deficiency and secondary hyperparathyroidism in addition to sodium thiosulfate, with significant improvements in her condition as seen in Figure 1.

## Conclusions

Non-uremic calciphylaxis presents as a rare and highly lethal disorder, prone to misdiagnosis as other ulcerative skin conditions. Such misidentification can yield grave consequences, both by postponing definitive treatment and by inadvertently exposing patients to additional calciphylaxis risk factors, such as systemic corticosteroids. Effective management demands a multidisciplinary strategy encompassing pain management, wound care, risk factor mitigation, and sodium thiosulfate therapy. To avert diagnostic delays in NUC, collaborative clinical efforts are paramount, ensuring precise histopathological diagnoses and tailored interventions. Swift recognition and interdisciplinary synergy remain pivotal in optimizing patient outcomes.
